# COGNITIVE HEALTH STATUS AND GENDER DIFFERENCES IN ATTITUDES AND EMOTIONS TOWARD OLDER ADULTS

**DOI:** 10.1093/geroni/igz038.1708

**Published:** 2019-11-08

**Authors:** Grace Caskie, Abigail R Voelkner, Hannah M Bashian

**Affiliations:** Lehigh University, Bethlehem, Pennsylvania, United States

## Abstract

The health and gender of older adults can elicit differing attitudes and emotions within young and middle-aged adults (Bergman & Bodner, 2015); one’s own gender may also influence these differences (Bergman & Cohen-Fridel, 2012). In this study, 287 participants (173 males, 114 females), aged 19-55 years (M=32.8), were randomly assigned to read one description of an older adult that varied cognitive health status (healthy/Alzheimer’s) and gender (male/female). Factorial MANOVAs examined differences by gender, health, and participant gender for participants’ (a) emotions about the older adult (compassion and emotional distance) and (b) negative perceptions about aging (ageist attitudes and aging anxiety). The first MANOVA found a significant main effect for health status; participants expressed more compassion (p=.013) and less emotional distance (p<.001) for the older adult with Alzheimer’s than for the healthy older adult. Also, the Target Gender X Participant Gender interaction was significant for emotional distance (p=.032), but not for compassion (p=.616); men reported more emotional distance than women for the female older adult, regardless of target health status, but men and women’s emotional distance were very similar for the male older adult. The second MANOVA showed only a significant health status main effect; ageist attitudes (p=.021), but not aging anxiety (p=.062), differed by health status of the older adult, with more ageist attitudes expressed for the healthy older adult than the older adult with Alzheimer’s. Overall, these results show that individual factors can influence young and middle-aged adults’ negative attitudes and emotions towards older adults. Implications will be discussed.

